# Discovery and characterization of a novel potent type II native and mutant BCR-ABL inhibitor (CHMFL-074) for Chronic Myeloid Leukemia (CML)

**DOI:** 10.18632/oncotarget.10037

**Published:** 2016-06-14

**Authors:** Feiyang Liu, Beilei Wang, Qiang Wang, Ziping Qi, Cheng Chen, Lu-Lu Kong, Ji-Yun Chen, Xiaochuan Liu, Aoli Wang, Chen Hu, Wenchao Wang, Huiping Wang, Fan Wu, Yanjie Ruan, Shuang Qi, Juan Liu, Fengming Zou, Zhenquan Hu, Wei Wang, Li Wang, Shanchun Zhang, Cai-Hong Yun, Zhimin Zhai, Jing Liu, Qingsong Liu

**Affiliations:** ^1^ High Magnetic Field Laboratory, Chinese Academy of Sciences, Hefei 230031, Anhui, P. R. China; ^2^ University of Science and Technology of China, Anhui, Hefei, 230036, P. R. China; ^3^ CHMFL-HCMTC Target Therapy Joint Laboratory, Hefei, Anhui, 230031, P. R. China; ^4^ Institute of Systems Biomedicine, Department of Biophysics, School of Basic Medical Sciences, Peking University Health Science Center, Beijing 100191, P.R. China; ^5^ Department of Chemistry, University of Science and Technology of China, Anhui, Hefei, 230036, P. R. China; ^6^ Department of Hematology, The Second Hospital of Anhui Medical University, Hefei, Anhui, 230601, P. R. China; ^7^ Hematology Research Center, Anhui Medical University, Hefei, Anhui 230601, P. R. China; ^8^ Hefei Cosource Medicine Technology Co. Ltd., Hefei, Anhui, 230031, P. R. China

**Keywords:** BCR-ABL, PDGFR, Chronic Myeloid Leukemia, kinase inhibitor

## Abstract

BCR gene fused ABL kinase is the critical driving force for the Philadelphia Chromosome positive (Ph+) Chronic Myeloid Leukemia (CML) and has been extensively explored as a drug target. With a structure-based drug design approach we have discovered a novel inhibitor CHMFL-074, that potently inhibits both the native and a variety of clinically emerged mutants of BCR-ABL kinase. The X-ray crystal structure of CHMFL-074 in complex with ABL1 kinase (PDB ID: 5HU9) revealed a typical type II binding mode (DFG-out) but relatively rare hinge binding. Kinome wide selectivity profiling demonstrated that CHMFL-074 bore a high selectivity (S score(1) = 0.03) and potently inhibited ABL1 kinase (IC_50_: 24 nM) and PDGFR α/β (IC_50_: 71 nM and 88 nM). CHMFL-074 displayed strong anti-proliferative efficacy against BCR-ABL–driven CML cell lines such as K562 (GI_50_: 56 nM), MEG-01 (GI_50_: 18 nM) and KU812 (GI_50_: 57 nM). CHMFL-074 arrested cell cycle into the G0/G1 phase and induced apoptosis in the Ph+ CML cell lines. In addition, it potently inhibited the CML patient primary cell's proliferation but did not affect the normal bone marrow cells. In the CML cell K562 inoculated xenograft mouse model, oral administration of 100 mg/kg/d of CHMFL-074 achieved a tumor growth inhibition (TGI) of 65% without exhibiting apparent toxicity. As a potential drug candidate for fighting CML, CHMFL-074 is under extensive preclinical safety evaluation now.

## INTRODUCTION

Chronic Myeloid Leukemia (CML) is a hematological cancer of the white cells and constitutes about 15% of adult leukemia with a diagnostic rate of 1 ∼ 2/100,000 people [1]. The pivotal driving force of the CML is the deregulation of the protein tyrosine kinase ABL1, which get constitutively expressed and activated due to genetic fusion of Abelson (ABL) gene with the chromosome 9 and 22 [t(9;22)(q34;q11)] of the break point cluster region (BCR) gene [2, 3]. Usually the CML patients express the 210 kDa BCR-ABL. The constitutive activation of the fused BCR-ABL kinase leads to the growth factors-independent proliferation and stimulate the downstream mediators such as STAT5, CRKL and ERK to remain the CML cells constitutive growth and survival [4]. The first BCR-ABL inhibitor, Imatinib (Gleevec [5]), as the seminal target therapy for the CML has achieved great clinical success and several other ABL inhibitors such as Nilotinib [6], Dasatinib [7], Bosutinib [8] and Ponatinib [9] have been developed to be used as the second line therapy to overcome a variety of the drug resistances induced by either various of point mutations or the chronic drug treatment induced BCR-ABL gene amplification [10]. In addition, a number of newly discovered inhibitors with different drug profile are under development now [11]. Given the fact that more and more drug-treatment-induced point mutations associated resistance emerged from the clinic and different drugs bear different efficacy and toxicity profiles, diverse pharmacophore-based new inhibitors are needed to provide more options for fighting the CML. Here we report a novel type II BCR-ABL kinase /PDGFR dual kinase inhibitor, CHMFL-074, which exhibits high selectivity among the kinome and high potencies against both the native and a variety of clinically important mutants of BCR-ABL kinases both *in vitro* and *in vivo*.

## RESULTS

### CHMFL-074 is a highly potent and selective BCR-ABL/PDGFR inhibitor

**Figure 1:**
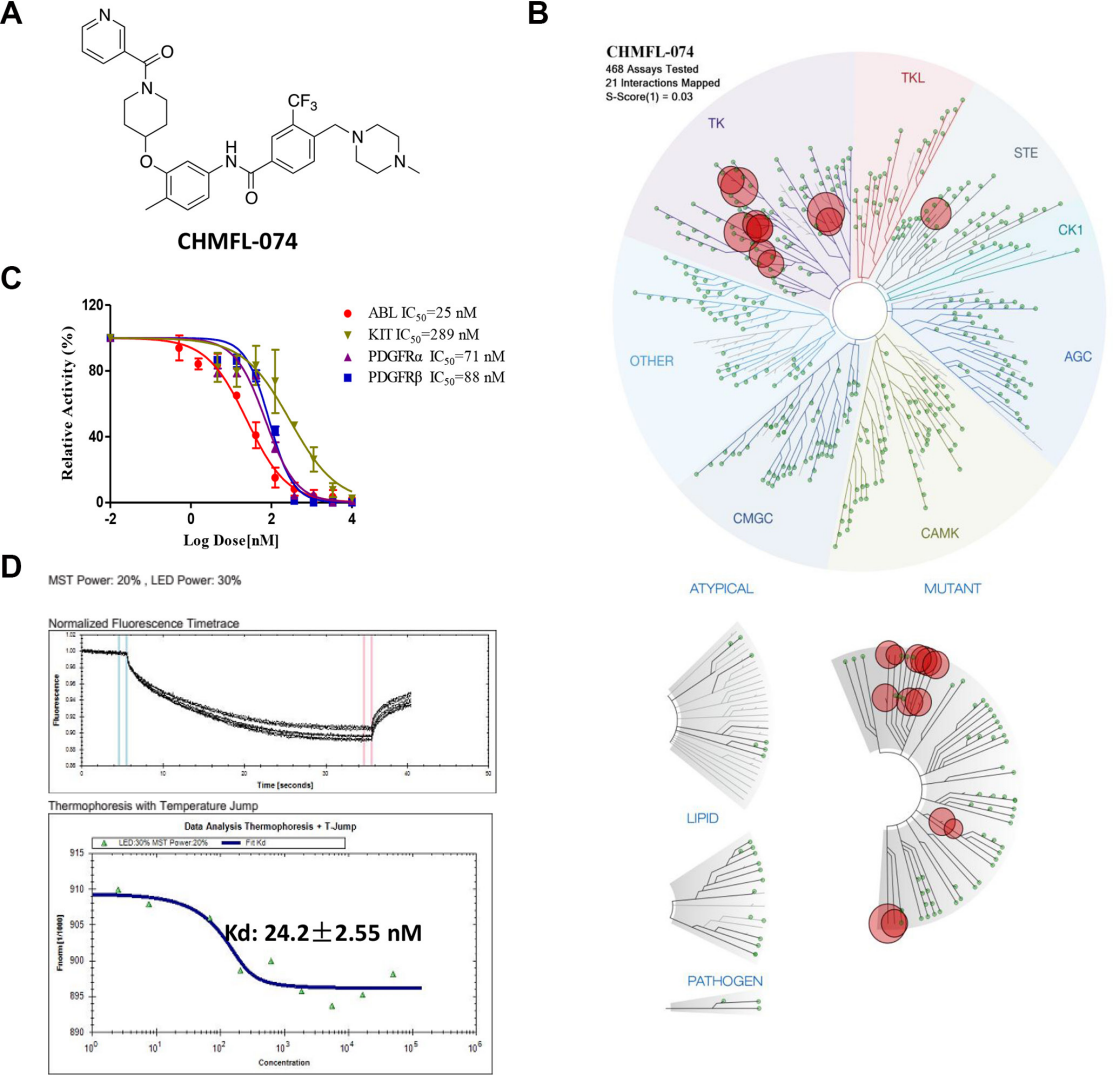
Characterization of CHMFL-074's biochemical activity and selectivity (**A**) Chemical structure of CHMFL-074. (**B**) DiscoverRx's KinomeScan^TM^ selectivity profiling of CHMFL-074 among 468 kinases and mutants. (**C**) Biochemical activity test of CHMFL-074 against ABL1, c-KIT and PDGFR kinases. (**D**) Microscale Thermophoresis Technology (MST) based binding Kd test of CHMFL-074 against ABL1 kinase.

Aiming to develop a highly selective BCR-ABL inhibitor, starting from the Imatinib core pharmacophore, we performed a focused medicinal library design and screening which led to the discovery of CHMFL-074 (Chemical Structure shown in Figure 1A). Examination of CHMFL-074's selectivity profile in the DiscoveRx's KinomeScan^TM^ platform at 1 μM concentration revealed a highly selective score (S-score (1) = 0.03) (Figure 1B and Supplementary Table 1). Besides native ABL1 kinase and a variety of clinically emerged mutants ABL1 mutants, such as ABL1 E255K, H396P, M351T, Q252H and Y253F, it also has strong binding affinity (% control number less than 1) against BLK, CSF1R, DDR1/2, LCK, LOK, PDGFRβ, and RET kinases (Supplementary Table 2). In order to further confirm these possible targets, we first used TEL-transfused BaF3 system to verify CHMFL-074's inhibitory activity against the ABL1 targets (Table 1). The results demonstrated that it potently inhibited the growth of p210 (wide type ABL1) dependent BaF3 cells but not parental BaF3 cells (GI_50_: 0.164 μM versus over 10 μM). In addition, it also potently inhibited ABL1's mutants such as E255K, F317L, F317I, M351T, Q252H, Y253F but weakly inhibited H396P and almost lost the activity against the gatekeeper mutation T315I. Interestingly, CHMFL-074 exhibited stronger inhibitory activity against ABL1 and related mutants than Imatinib. We then tested a panel of other kinases that showed strong binding affinity in the KinomeScan^TM^ profiles in the TEL or BCR transfused BaF3 assay system. The results demonstrated that CHMFL-074 did not display strong inhibitory activity against BLK, LCK, DDR1/2 but weakly inhibited RET kinase (Table 1). However, it did potently inhibit PDGFRα/β kinase activity (GI_50_: 0.095 μM and 0.052 μM respectively), though it was less active than Imatinib against them. These data suggested that CHMFL-074 might be a potent BCR-ABL and PDGFR dual kinase inhibitor. Further confirmation with Invitrogen Z'LYTE biochemical assay (activity-based assay) revealed an IC_50_ of 25 nM against ABL1 kinase (Figure 1C). The Microscale Thermophoresis Technology (MST) based assay (binding affinity assay) revealed a binding Kd of 24 nM (Figure 1D). In the Promega's ADP-Glo biochemical assay (activity-based assay), it displayed IC_50_s of 71 nM and 88 nM against PDGFRα and β respectively (Figure 1C). In addition, since Imatinib also potently inhibited c-KIT kinase, we also tested CHMFL-074 against c-KIT. The Z'LYTE assay data showed that it was about 12-fold less active than ABL1 kinase (IC_50_: 289 nM).

**Table 1:** Anti-proliferation effect of CHMFL-074 against a panel of BaF3 isogenic cell lines

Cell lines	Imatinib GI_50_: (μM)	CHMFL-074 GI_50_: (μM)
BaF3	> 10	> 10
P210-BaF3	0.27	0.164
P210-E255K-BaF3	1.93	0.774
P210-F317L-BaF3	2.16	0.218
P210-F317I-BaF3	0.85	0.355
P210-M351T-BaF3	0.625	0.349
P210-Q252H-BaF3	0.659	0.118
P210-Y253F-BaF3	> 10	0.338
P210-H369P-BaF3	1.79	1.04
P210-T315I-BaF3	> 10	> 10
Tel-BLK-BaF3	> 10	2.86
Tel-RET-BaF3	> 10	0.63
Tel-LCK-BaF3	> 10	2.02
Tel-HCK-BaF3	> 10	> 10
Tel-PDGFRα-BaF3	0.034	0.095
Tel-PDGFRβ-BaF3	0.019	0.052
TEL-DDR1-BaF3	9.43	3.52
BCR-DDR2-BaF3	> 10	2.67

### CHMFL-074 adopts a distinct binding mode against BCR-ABL kinase

**Figure 2:**
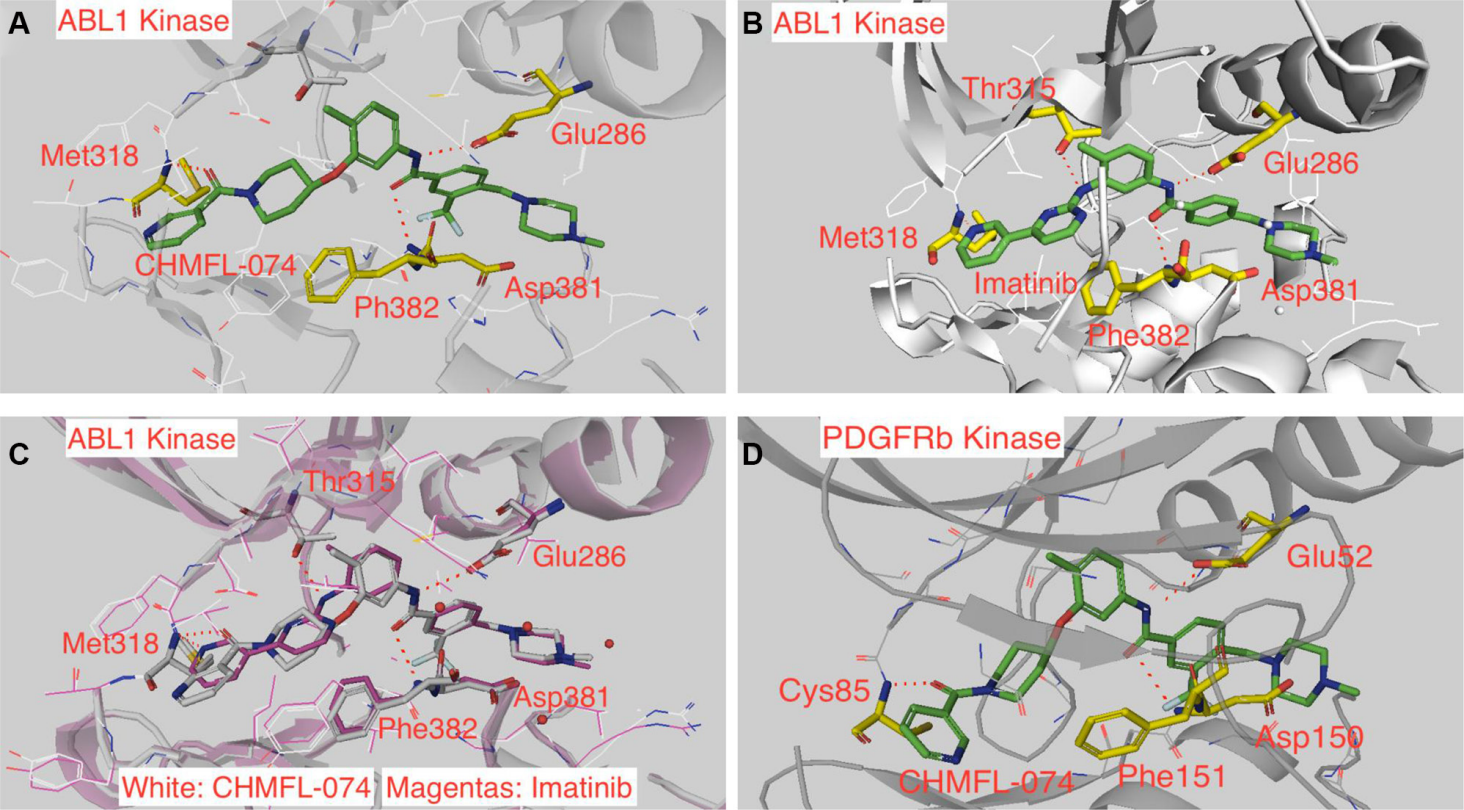
CHMFL-074's binding mode in ABL1 and PDGFRβ kinases (**A**) Crystal structure of CHMFL-074 in complex with ABL1 kinase (PDB ID: 5HU9). (**B**) Crystal structure of Imatinib in complex of ABL1 kinase (PDB ID: 2HYY). (**C**) Superimposition of CHMFL-074 and Imatinib in the ABL1 kinase (PDB ID: 5HU9 and 2HYY). (**D**) CHMFL-074 docked into homology model of PDGFRβ kinase (built upon PDB ID: 1T46).

In order to understand the binding mechanism of CHMFL-074 against ABL1 kinase, we then tried to crystalize CHMFL-074 with ABL1 kinase and fortunately we obtained a high resolution (1.53 Å) crystal structure (PDB ID: 5HU9 and Supplementary Table 3) (Figure 2A). The results demonstrated that CHMFL-074 adopted a typical type II binding mode (DFG-out conformation) to ABL1, which was represented by two canonical hydrogen bonds formed between the Glu286 located in the c-Helix and Asp381 located in the DFG motif with the amide bond (NHC=O) in the drug. This is similar with Imatinib which also binds to ABL1 kinase with type II binding mode (PDB ID: 2HYY) (Figure 2B). However, superimposition of CHMFL-074 with Imatinib revealed a distinct hinge binding. CHMFL-074 used oxygen atom of carbonyl group connecting the terminal pyridine and piperidine ring to form the hinge binding hydrogen bonds with Met318, while Imatinib used the terminal pyridine ring's N atom to form this hinge binding (Figure 2C). In addition, Imatinib formed an extra hydrogen bond between the aminopyrimidine's NH in the drug with the gatekeeper residue Thr315. However, CHMFL-074 used O-linked piperidine ring to replace this aminopyrimidine ring and abolished this hydrogen bond. Docking results of CHMFL-074 with a homology model of PDGFRβ kinase (built upon PDB ID: 1T46) revealed a similar binding mode with ABL1 kinase (Figure 2D). It is noteworthy that most of the type II inhibitors used the nitrogen atoms in the aromatic heterocyclic to form the hinge binding, and oxygen atom in the carbonyl group to serve as the hinge binding hydrogen donor is relatively rare.

### CHMFL-074 exhibits selective and potent anti-proliferative effect against BCR-ABL dependent CML cell lines and patient primary cells

**Figure 3:**
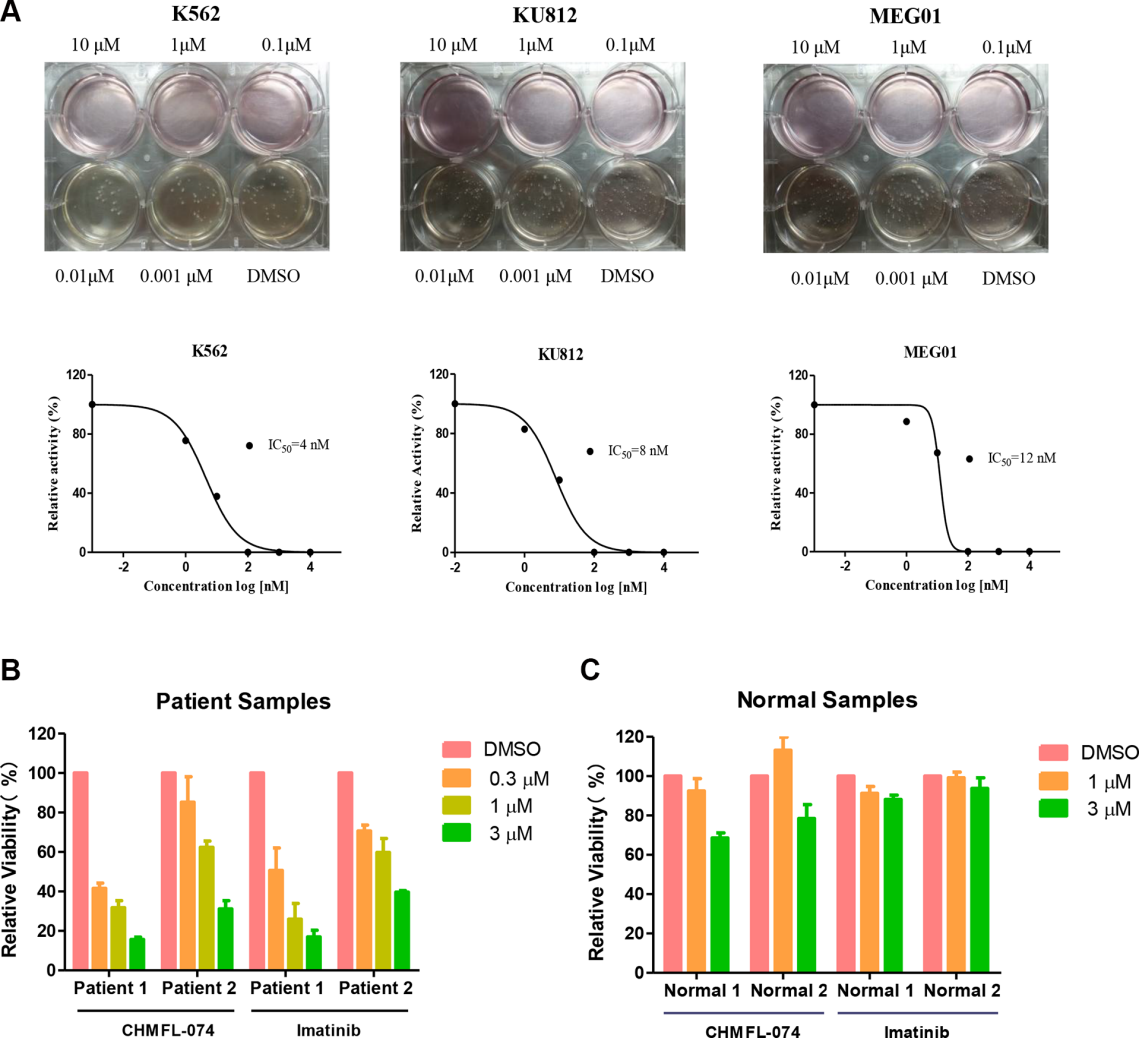
CHMFL-074's anti-colony formation effects against intact cancer cell lines and anti-proliferative effect against CML patient primary cells (**A**) CHMFL-074's anti-colony formation effect against K562, KU812 and MEG-01 cells. (**B**) CHMFL-074's anti-proliferative effect against BCR-ABL positive CML patient primary cells and (**C**) normal white cells purified from the peripheral blood.

We next tested CHMFL-074 in a panel of leukemia cell lines. Not surprisingly, it exhibited strong anti-proliferative effect against BCR-ABL dependent CML cell lines such as K562 (GI_50_: 0.056 μM), KU812 (GI_50_: 0.057 μM) and MEG-01 (GI_50_: 0.018 μM) (Table 2). In general, CHMFL-074 was 3–5 fold more potent than Imatinib against these cells. As expected, both CHMFL-074 and Imatinib did not display apparent anti-proliferative effect against other leukemia cell lines such as AML cells U937 and HL-60, MCL cell line REC-1 and CLL cell line MEC-1. CHMFL-074 also did not inhibit the growth of the normal Chinese hamster ovary cells indicating a non-general cytotoxicity. In addition, CHMFL-074 potently blocked the colony formation of BCR-ABL dependent cell lines K562 (EC_50_: 4 nM), KU812 (EC_50_: 8 nM) and MEG-01 (EC_50_: 12 nM) (Figure 3A). In the BCR-ABL positive CML patient primary cells, CHMFL-074 exhibited half effective inhibitory concentrations between 0.3–1 μM which followed similar trend as Imatinib (Figure 3B). Meanwhile, this drug did not show any inhibitory activities against normal human B-cells purified from the peripheral blood at 1 μM (Figure 3C).

**Table 2:** Anti-proliferative effect of CHMFL-074 against a panel of intact cancer cell lines

Cell lines	Imatinib GI_50_: (μM)	CHMFL-074 GI_50_: (μM)
K562	0.267	0.056
KU812	0.163	0.057
MEG-01	0.074	0.018
U937	> 10	10
HL-60	> 10	> 10
REC-1	> 10	> 10
MEC-1	> 10	> 10
CHO	> 10	> 10

### CHMFL-074 blocks BCR-ABL mediated signaling pathways and arrests cell cycle

**Figure 4:**
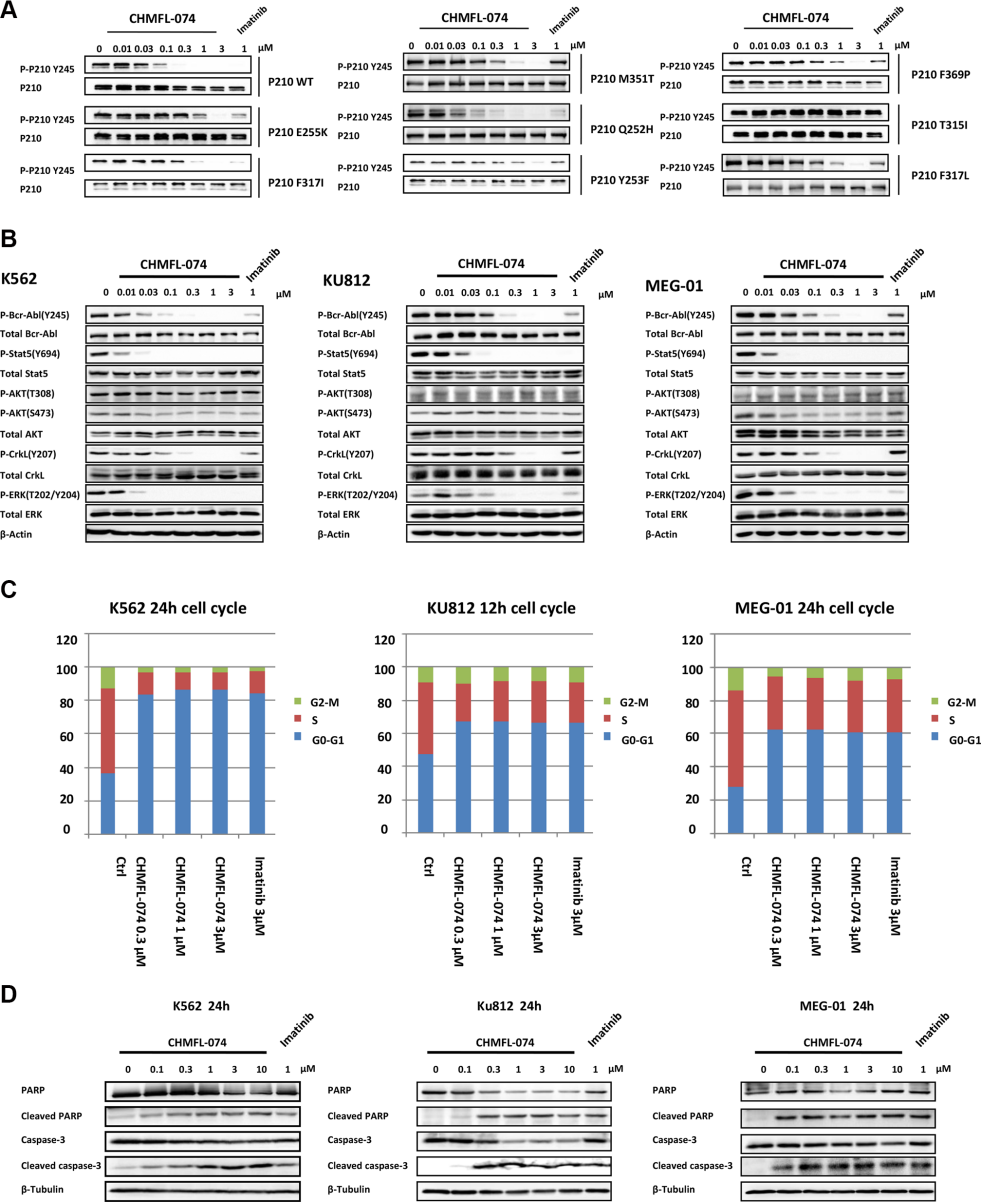
CHMFL-074's effect on BCR-ABL mediated signaling pathway, cell cycle progression and apoptosis (**A**) CHMFL-074's effect on BCR-ABL(p210) Y245 phosphorylation site in the native and mutants of p210 transformed BaF3 isogenic cell lines. (**B**) CHMFL-074's effect on BCR-ABL mediated signaling pathways in the K562, KU812 and MEG-01 cells at different concentrations at 2 h. (**C**) CHMFL-074's effect on the cell cycle progression in the K562 (24 h), KU812 (12 h) and MEG-01(24 h) cells at different concentrations. (**D**) CHMFL-074's effect on the apoptosis induction at different concentrations in K562, KU812 and MEG-01 cells at 24 h.

We then examined CHMFL-074's effect in the BCR-ABL mediated signaling pathways. In the engineered p210 native and mutants isogenic BaF3 cells, it potently inhibited phosphorylation of Y245 site in BCR-ABL fusion protein in the wide type, E255K, F317I, F317L, M351T, Q252H, Y253F, and F369P, but did not affect T315I mutants, which is in accordant with the anti-proliferative results (Figure 4A). The results demonstrated that it potently inhibited the BCR-ABL's auto-phosphorylation at Y245 site in the K562 cells (EC_50_: < 100 nM) and displayed better inhibitory activities than Imatinib (Figure 4B left panel). CHMFL-074 also significantly blocked downstream signaling mediators such as pStat5, pCrkL and pERK in K562 cells, following the trend of better inhibition than Imatinib. Similar results were observed in the other two BCR-ABL dependent CML cell lines KU812 and MEG-01, confirming that this drug has strong inhibitory effect in the BCR-ABL mediated signaling pathways (Figure 4B middle and right panel). In addition, starting from 0.3 μM concentration, CHMFL-074 could effectively induce G0/G1 phase cell cycle arrest and displayed a similar effect with Imatinib at 3 μM in K562, KU812 and MEG-01 cells (Figure 4C). Apparent dose-dependent apoptosis was observed by examination of the cleavage PARP and Caspase-3 of these cell lines (Figure 4D).

### CHMFL-074 suppresses tumor progression in the K562 cell inoculated mouse model

**Figure 5:**
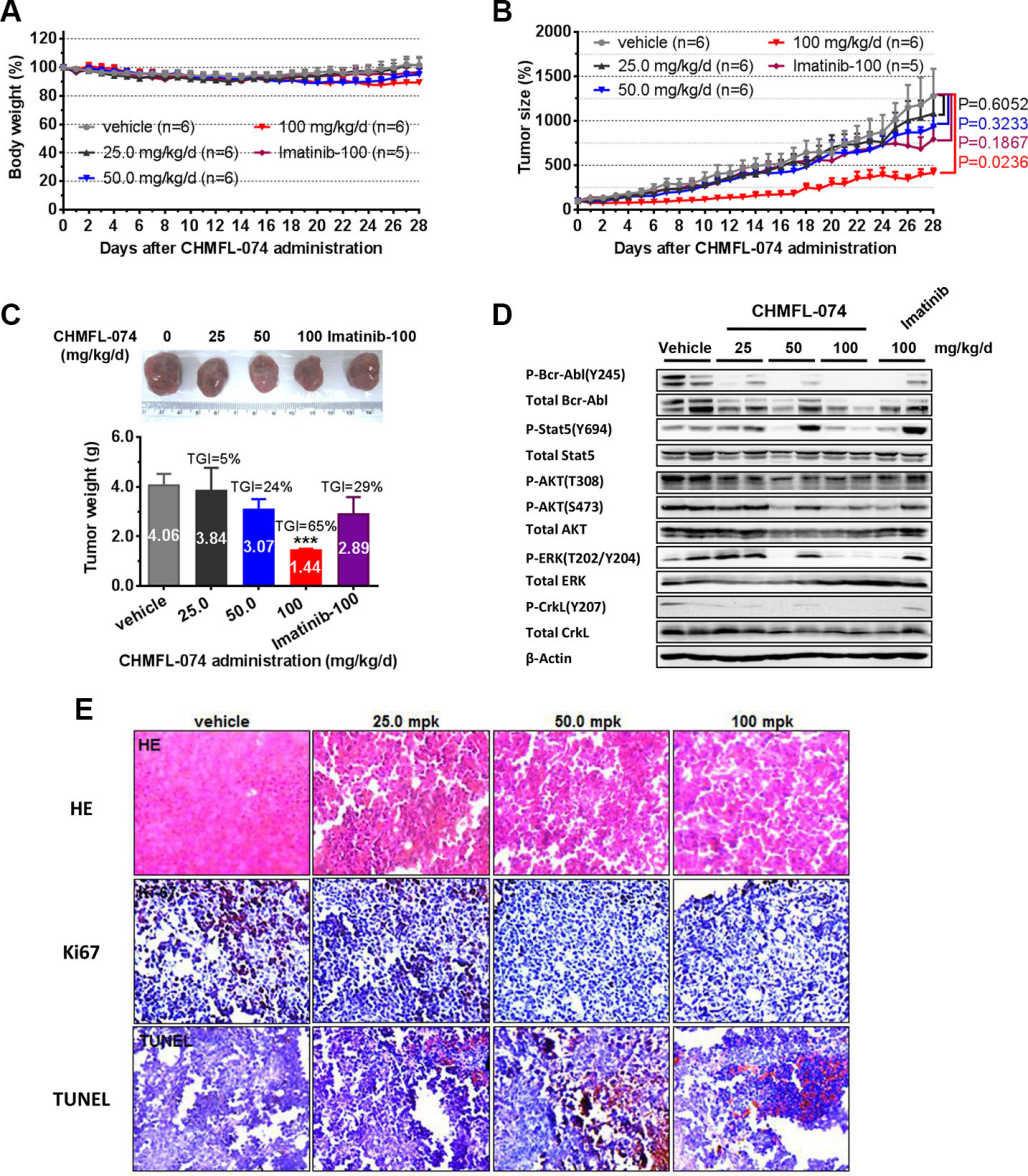
CHMFL-074's anti-tumor efficacy in K562 xenograft model Female nu/nu mice bearing established K562 tumor xenografts were treated with CHMFL-074 at 25.0, 50.0, 100 mg/kg/d, or vehicle. Imatinib at 100 mg/kg/d served as positive control. Daily oral administration was initiated when K562 tumors had reached a size of 200 to 300 mm^3^. Each group contained 6 animals. Data, mean ± SEM. (**A**) Body weight and (**B**) tumor size measurements from K562 xenograft mice after CHMFL-074 administration. Initial body weight was set as 100%. Non-paired Student's *t*-test was used to compare the means of two groups and *P* < 0.05 was considered statistically significant. (**C**) Representative photographs of tumors in each group after 25.0, 50.0 or 100 mg/kg/d CHMFL-074 or vehicle treatment. Imatinib at 100 mg/kg/d served as positive control (upper panel). Comparison of the final tumor weight in each group after 12-day treatment period. Numbers in columns indicate the mean tumor weight in each group. ns, *P* > 0.05, *P < 0.05, **P < 0.01 (lower panel). (**D**) Western blot analysis with antibodies specific to the indicated proteins from tumor lysates prepared from the K562 xenograft mice upon the completion of the indicated treatments. β-Actin is shown as the loading control. (**E**) Tumor tissue histology of serial section of tumor-bearing mice from each group. Data shows HE, Ki-67 and TUNEL staining. Brown staining indicates positive cells. (Original magnification 200×).

The pharmacokinetic profile study of CHMFL-074 in the Sprague-Dawley rats demonstrated that it bore an acceptable PK (T_1/2_ 3.38 h, Bioavailability 42.93%) for the oral administration (Table 3). In K562 inoculated mouse model, oral administration of CHMFL-074 exhibited dose-dependent tumor progression suppression efficacy and 100 mg/kg/day dosage could almost block the tumor growth with a TGI (tumor inhibition rate) of 65% while no apparent toxicity was observed (Figure 5A–5C). As expected, reduced phosphorylation of Bcr-Abl and related downstream mediators such as STAT5, ERK, CRKL in tumors was observed compared to that in vehicle-treated controls (Figure 5D). Immunohistochemical (IHC) staining showed that CHMFL-074 dose dependently inhibited the cell proliferation (Ki67 stain) and induced apoptosis (TUNEL stain) (Figure 5E).

**Table 3:** Pharmacokinetic study of CHMFL-074 in Sprague-Dawley rat

	t_1/2_	T_max_	C_max_	AUC_(0-t)_	AUC_(0-∞)_	Vz	CLz	MRT_(0-∞)_	F
	hr	hr	ng/mL	ng/mL*hr	ng/mL*hr	mL/kg	mL/hr/kg	hr	%
IV 1mg/kg									
Mean	1.23	0.02	739.18	336.71	339.92	5254.46	2959.65	1.29	NA
SD	0.05	0.00	96.24	31.44	31.97	546.04	284.23	0.05	NA
PO 10 mg/kg									
Mean	3.38	4.00	190.12	1126.41	1459.30	NA	NA	6.58	42.93
SD	0.41	2.00	66.82	407.72	685.82	NA	NA	0.43	NA

## DISCUSSION

Imatinib, the first launched target therapy, which potently inhibits BCR-ABL, c-KIT and PDGFRs has achieved remarkable success in the clinic [5]. However, after chronic drug treatment, most patient relapse due to the several different resistance mechanisms. One of the most prevalent resistance mechanisms is due to the over 30 different point mutations, including G250E, Y253H, E255K, F317L, M361T and the critical gatekeeper T315I mutation, which decreased the inhibitory activity of Imatinib [6]. The other important resistance mechanism is due to the amplification of the BCR-ABL gene [10]. A number of new drugs have been developed which either/both increased the activity against native BCR-ABL kinase or/and the variety of the mutants. The different pharmacophore-based drugs displayed different efficacy profiles against these number of mutants and there is still an unmet clinical need to develop new pharmacophore-based drugs which bear different efficacy profiles and serve as supplementary to the current anti-CML armory library.

CHMFL-074 displays better inhibitory activity than Imatinib against native BCR-ABL kinase and a number of mutations such as E255K, F317L, F317I, M351T, Q252H,Y253F and H369P. However, it did not inhibit the critical gatekeeper mutant T315I. The relatively rare binding mode of CHMFL-074, which uses carbonyl oxygen as the hinge binding, also highlights the new features of this type II compound. In summary, the data presented here showed that CHMFL-074 was highly effective against both the intact CML cell lines and BCR-ABL positive patient primary hematopoietic cells. It exhibited certain advantages over Imatinib regarding the stronger potencies and capability to inhibit some of the Imatinib resistant mutations. The acceptable PK profile and *in vivo* efficacy again provided additional rationale to further develop this compound as a supplementary to the current anti-BCR-ABL positive CML therapy.

## MATERIALS AND METHODS

### Inhibitors

CHMFL-074 was synthesized in the lab (procedure following patent: CN201410757626.7) and dissolved in DMSO at a stock concentration of 10 mM and stored in aliquots at −20°C. Imatinib was purchased from Shanghai Haoyuan Chemexpress Inc. (Shanghai, China).

### Cell lines and cell culture

The K562 (CML), KU812 (CML), MEG-01 (CML), MV4-11 (AML), MOLM14 (AML), U937 (AML), REC-1 (Human B-cell lymphoma cell), HL-60 (Human promyelocytic leukemia cells), MEC-1(CLL), CHL (Hamster lung cell), CHO (Hamster ovary cell) cell lines were obtained from American Type Culture Collection (Manassas, VA). All the cells were grown in a humidified incubator (Thermo, USA) at 37°C under 5% CO_2_. CHO cells were maintained in DMEM supplemented with 10% FBS, 1% penicillin/strepto-mycin. MV4-11 (AML) were grown in IMDM supplemented with 10% FBS, 1% penicillin/streptomycin. All the other cell lines and all the isogenic BaF3 cells were grown in RPMI 1640 medium supported with 10% FBS, and 1% penicillin/streptomycin. Adherent cells were grown in tissue culture flasks until they were 85–95% confluent prior to use. For suspension cells, cells were collected by spinning down at 800 rpm/min for 5 min before use.

### Immunoblotting

K562, KU812, MEG-01cells were treated with DMSO, serially diluted compound CHMFL-074, 1 μM Imatinib for 2 h before immunoblotting. Cells were washed with ice cold phosphate buffered saline (PBS), lysed using radio-immunoprecipitation (RIPA) buffer [150 mM NaCl, 1% (vol/vol) Nonidet P-40, 0.5% (wt/vol) sodium deoxycholate, 0.1% (wt/vol) SDS] in 50 mM Tris HCl (pH 8.0) supplemented with protease and phosphatase inhibitors (Thermo, USA). Protein concentrations were determined using the BCA Protein Assay kit (Beyotime, China) according to the manufacturer's protocol. Proteins were separated by SDS-PAGE and transferred to an Immobilon-P PVDF membrane (Millipore, USA), and blocked in 5% dry milk in Tris Buffered Saline, with Tween 20 (TBST). Membranes were incubated with primary and secondary antibodies, and target proteins were detected with ECL detection reagent (Pierce, USA). β-Actin (Sigma-Aldrich) served as a loading control. Phospho-c-Abl (Tyr245)(73E5) Rabbit mAb (2868), c-Abl antibody (2862), STAT5 (3H7) Rabbit mAb (9358), Phospho-STAT5 (Tyr694)(C71E5) Rabbit mAb (9314), Akt (pan)(C67E7) Rabbit mAb (4691), Phospho-Akt (Thr308) (244F9) Rabbit mAb (4056), Phospho-Akt (Ser473) (D9E) XP^®^ Rabbit mAb (4060), Phospho-CrkL (Tyr207) antibody (3181), CrkL (32H4) Mouse mAb (3182), Phospho-p44/42 MAPK (Erk1/2) (Thr202/Tyr204) (197G2) Rabbit mAb (4377), p44/42 MAPK (Erk1/2) (137F5) Rabbit mAb (4695) antibodies were obtained from Cell Signaling Technology (MA, USA).

### ABL1 and c-KIT protein purification

A construct encoding c-ABL residues 229–500 with a His tag was cloned into baculovirus expression vector pFASTHTA. The protein was expressed by infecting SF9 cells with high titer viral stocks for 48 hours. Cells were harvested and lysed in 30 mM Tris pH 7.4, 150 mM NaCl, 3 mM KCl, 10% glycerol, 1 mM PMSF, 2 mM TCEP, 1 mM ADP, 20 mM Imidazole. The supernatant was loaded to Ni-NTA Column (QIAGEN, 1018244). Then the proteins were gradient washed using the same buffer with 50 mM, 100 mM imidazole, then the ABL protein was eluted with Elution buffer (20 mM Tris, 500 mM NaCl, 1% glycerol, 1 mM TCEP, 0.5 mM ADP, 300 mM Imidazole, pH 8.0). The eluted protein was loaded on desalt column PD-10(GE) to change the buffer to 20 mM Tris, 500 mM NaCl, 1% glycerol, 2 mM TCEP, pH 8.0. The protein was concentrated to 1 mg/ml and aliquots were frozen and stored at −80^°^C.

### Kinase biochemical assay

The fluorescence resonance energy transfer-based Z′-LYTE kinase assay (Invitrogen, USA) was used to evaluate the IC_50_ value of CHMFL-074 for inhibition of ABL and KIT kinase. The reaction was performed on a 384-well plate with a 10 μL reaction volume per well containing 2 μM peptide (Tyr 02 peptide for ABL kinase, Tyr 06 peptide for KIT kinase) substrate in reaction buffer, and ABL kinase (2.5 μL, 5 ng) or KIT kinase (2.5 μL, 10 ng) with a serial 3-fold dilution of CHMFL-074 (2.5 μL, 10 μM to 1.5 nM). The final reaction concentration of ATP was 300 μM. After 1 hour incubation, the reaction was developed and terminated, and the fluorescence measured with an automated plate reader (SpectraMax I3, USA). A dose-response curve was fitted using Prism 5.0 (GraphPad Software Inc., San Diego, CA).

The ADP-Glo™ kinase assay (Promega, USA) was used to screen CHMFL-074 for its PDGFRα/PDGFRβ inhibition effects. The kinase reaction system contains PDGFRα (9 μL, 90 ng), PDGFRβ (9 μL, 90 ng), 1 μL of serially diluted CHMFL-074, and 10 μL substrate Poly (4:1 Glu, Tyr) peptide (0.4 μg/μL) (Promega, USA) with 100 μM ATP (Promega, USA). The reaction in each tube was started immediately by adding ATP and kept going for an hour under 37°C. After the tube cooled for 5 minutes at room temperature, 5 μL solvent reactions were carried out in a 384-well plate. Then 5 μL of ADP-Glo™ reagent was added into each well to stop the reaction and consume the remaining ADP within 40 minutes. At the end, 10 μL of kinase detection reagent was added to the wells and incubated for 30 minutes to produce luminescence signal. Luminescence signal was measured with an automated plate reader (Envision, USA) and the dose-response curve was fitted using Prism 5.0 (GraphPad Software Inc., San Diego, CA).

### Microscale thermophoresis (MST) binding Kd assay

The Kd was measured using the Monolith NT.115 from Nanotemper Technologies. Proteins were fluorescently labeled according to the manufacturer's protocol. A range of concentrations of ligands CHMFL-074 and Imatinib (range from 0.05 mM to 2.5 nM) were incubated with 200 nM of purified Abl protein 5 min in assay buffer (20 mM Tris, 150 mM NaCl, pH 8.0, and 0.05% Tween 20). The sample was loaded into the NanoTemper glass capillaries and microthermophoresis carried out using 20% LED power and 30% MST Power. KD were calculated using the mass action equation via the NanoTemper software from duplicate reads of triplicate experiments.

### Protein preparation and crystallization

Construct of ABL1 kinase domain spanning residues 229–500 was PCR-cloned from human cDNA library and inserted into pFastBac-HTB plasmid between the *Bam* HI and *Xho* I restriction sites. The N-terminal 6xHis-tagged ABL1 protein (a TEV protease cleavage site was inserted between the tag and ABL1 for later removal of the tag) was expressed in Sf9 insect cells. After harvesting, the cells were broken by sonication in lysis buffer (30 mM Tris, 150 mM NaCl, 3 mM KCl, 10% glycerol, 1 mM PMSF, 1 mM TCEP, 20 mM Imidazole, pH 7.4). Cell lysate was obtained by centrifugation at 20,000 rpm for 1 hour at 4°C and then applied to the Chelating Sepharose Beads (GE Healthcare, Cat. 17-0575-02) charged with Ni^2+^. The beads were thoroughly washed with wash buffer (20 mM Tris, 500 mM NaCl, 1% glycerol, 0.5 mM TCEP, 30 mM Imidazole, pH 8.0), and then the protein was eluted with elution buffer (20 mM Tris, 500 mM NaCl, 1% glycerol, 1 mM TCEP, 300 mM Imidazole, pH 8.0). The eluent was concentrated and cleaved by TEV enzyme to remove the His-tag, and then applied to gel-filtration chromatography using a Superdex 200 column (GE Healthcare, Cat. 17-5175-01) to further purify the ABL1 protein. The purified protein was concentrated and dispensed into aliquots, flash-frozen in liquid nitrogen and stored in −80°C freezer for later using.

For co-crystallization, 100 mM CHMFL-074 solution was added to 16 mg/mL ABL1 protein solution to achieve the final concentration of 1 mM compound. The mixture was incubated on ice for 0.5 hours and then applied to crystallization using the hanging drop vapor diffusion method. The crystallization reservoir solution was 0.1 M Potassium thiocyanate, 28% PEG2000MME.

### Crystal structure determination and refinement

Diffraction data of the ABL1/CHMFL-074 crystal were collected at 100 K on beamline BL17U1 at Shanghai Synchrotron Radiation Facility (SSRF). The diffraction data were processed using HKL-3000. The structure was solved by molecular replacement with Phaser using the previously reported ABL1 structure (PDB ID 2HYY) as the search model. CNS was then used to obtain a less biased model (by simulated-annealing) and calculate the sigmaA weighted 2Fo-Fc and Fo-Fc maps for manual inspection and adjustment. Repeated rounds of manual refitting and crystallographic refinement were then performed using COOT and Phenix. The inhibitor was modeled into the closely fitting positive Fo-Fc electron density and included in following refinement cycles. Topology and parameter files for the inhibitor were generated using PRODRG. The crystal diffraction data and refinement statistics were summarized in Supplementary Table 3. The refined ABL1/CHMFL-074 crystal structure was deposited in Protein Data Bank with the entry ID 5HU9.

### Molecular modeling of PDGFRβ kinase

Currently there are no crystal structure for the kinase domain of PDGFRα/β in the PDB, homology models were built using *MODELLER* 9.15 [12] for docking and comparison. The cKIT kinase domain (PDB ID: 1T46) was used as the template, the sequence alignment was shown in Supplementary Figure 1, the lowest-energy models generated by default settings were further refined using *AmberTools* [13]. CHMFL-074 was constructed using Bio^X^ 4.6 [14] and manually docked into the ATP binding pocket of PDGFRα/β using Yeti^X^ 8.3 [15], the side chain flexibility [16] and directional hydrogen bonds [17] are considered during docking.

### BaF3 isogenic cell line generation

Retroviral constructs for fusion kinases were made based on the pMSCVpuro (Clontech) backbone. For TEL fusion vectors, the first 1 kb of human TEL gene with an artificial myristoylation sequence (MGCGCSSHPEDD) was cloned into pMSCVpuro retroviral vector, followed by a 3xFLAG tag sequence and a stop codon; for BCR fusion vectors, the first 2.8 kb coding region of p210 amplified from K562 cell line was used in fusion constructs. Then the kinase domain coding sequences were inserted in-frame between TEL/BCR and 3xFLAG sequences. All mutagenesis were performed using the QuikChange Site-Directed Mutagenesis Kit (Stratagene) following the manufacturer's instructions. Retrovirus was packaged in HEK293T cells by transfecting kinase-fusion MSCV vectors together with two helper plasmids, virus supernatants were harvested 48 hours after transfection and filtered before infection. Then BaF3 cells were infected with harvested virus supernatants using spinoculation protocol and stable cell lines were obtained by puromycin selection for 48 hours. A second selection in the absence of IL-3 was performed to obtain IL-3 independent cell lines that solely depend on the introduced kinase activities for cell proliferation.

### Anti-Proliferation studies

A density of 1 × 10^4^ to 2.5 × 10^4^ cells/mL cells were mixed with various concentrations of compounds then 100 μL suspension was added to each well and incubated for 72 hours. Cell viability was determined using the CellTiter-Glo assay (Promega, USA) or CCK-8 assay (Beboy, China). Both assays were performed according to the manufacturer instructions. For CellTiter-Glo assay, luminescence was determined in a multi-label reader (Envision, PerkinElmer, USA). For CCK-8 assay, absorbance was measured in a microplate reader (iMARK, Bio-Rad, USA) at 450 nm and 655 nm. Data were normalized to control group (DMSO). GI_50_ were calculated using Prism 6.0 (GraphPad Software, San Diego, CA).

### Patient primary cell proliferation assay

Mononuclear cells were isolated from CML patients or healthy donors by density-gradient centrifugation through Ficoll (Beijing Solarbio Science & Technology Co., Ltd., Beijing, China) at 400 g for 30 min, followed by two washes in 1× phosphate-buffered saline. Cells were then cultured in liquid culture (RPMI1640, supplemented with 20% FBS). All blood samples from CML patients and healthy donors were obtained through written consent under approval of the Chinese Academy of Sciences Institutional Review Board. The ethics committees approved the consent Procedure. CML primary patient cells were tested in liquid culture (RPMI1640, supplemented with 20% FBS) in the presence of different concentrations of CHMFL-074. The trypan blue exclusion assay was used for quantification of cells for seeding for before Cell-Titer Glo assays (Promega, USA). The Cell-Titer Glo assay was used for proliferation studies and carried out according to manufacturer's instructions. Cell viability is reported as percentages of control (untreated) cells.

### Cell cycle analysis

K562, KU812 and MEG01 cells were treated with serially diluted CHMFL-074 for 12 or 24 hours. The cells were fixed in 70% cold ethanol and incubated at −20°C overnight, then stained with PI/RNase staining buffer (BD Pharmingen). Flow cytometry was performed using a FACS Calibur (BD), and results were analyzed by ModFit software.

### Apoptosis effect examination

K562, KU812, MEG-01 cells were treated with DMSO, serially diluted CHMFL-074, 1 μM Imatinib for the indicated periods. Cells were collected and analyzed by Western blotting using following antibodies: PARP(9532), Caspase-3(9665) from Cell Signaling Technology(MA, USA). β-Actin (Sigma-Aldrich) served as a loading control.

### Colony formation assay

In brief, 1 mL of 3% agarose combined with 1 mL KU812, MEG01, or K562 growth media was used as the bottom agar in a 6-well plate. 800 cells in 1.7 mL growth media was combined with 0.3 ml of 3% agarose solution and plated on top of the bottom layer. Cells were maintained in a humidified 5% CO_2_ incubator at 37°C for 15 days, and continuously treated with serially diluted CHMFL-074 in a soft agar medium. On the 15th day, the number of colonies in each well were counted and each measurement was performed in triplicate.

### *In vivo* pharmacokinetics study

CHMFL-074 was dissolved in 55% saline containing 5% DMSO and 40% PEG400 by vortex. The final concentration of the stock solution was 1 mg/mL for administration. Six-eight weeks old male Sprague-Dawely rats were fasted overnight before starting drug treatment via intravenous (1 mg/kg) and oral administration (10 mg/kg). Animal blood collection time points were as follows: for group 1, 3, 5 (intravenous): 1 min, 5 min, 15 min, 30 min, 1 h, 2 h, 4 h, 6 h, 8 h before and after administration was selected; for group 2, 4, 6 (oral): 5 min, 15 min, 30 min, 1 h, 2 h, 4 h, 6 h, 8 h and 24 h before and after dosing. Each time about 0.2 mL blood was collected through the jugular vein adding heparin for anticoagulation and kept on ice. Then plasma was separated by centrifugation at 8000 rpm for 6 minutes at 2–8°C. The obtained plasma was stored at −80°C before analysis. After finishing the test, all surviving animals will be transferred to the repository or euthanasia (CO_2_ asphyxiation).

### K562 xenograft tumor model

Five-week old female nu/nu mice were purchased from the Shanghai Experimental Center, Chinese Science Academy (Shanghai, China). All animals were housed in a specific pathogen-free facility and used according to the animal care regulations of Hefei Institutes of Physical Science Chinese Academy of Sciences. Prior to implantation, cells were harvested during exponential growth. Five million K562 cells in PBS were formulated as a 1:1 mixture with Matrigel (BD Biosciences) and injected into the subcutaneous space on the right flank of nu/nu mice. Daily oral administration was initiated when K562 tumors had reached a size of 200 to 300 mm^3^. Animals were then randomized into treatment groups of 6 mice each for efficacy studies. CHMFL-074 was delivered daily in a HKI solution (0.5% Methocellulose/0.4% Tween 80 in dd H_2_O) by orally gavage. A range of doses of CHMFL-074 or its vehicle were administered, as indicated in figure legends. Body weight was measured daily and tumor growth was measured every day after CHMFL-074 treatment. Tumor volumes were calculated as follows: tumor volume (mm^3^) = [(W^2^ × L)/2] in which width (W) is defined as the smaller of the two measurements and length (L) is defined as the larger of the two measurements.

### Histological examination

Tumor tissues were fixed in 10% neutral-buffered formalin and embedded in paraffin. Six-micron tissue section were prepared, deparaffinized, dehydrated, and then stained with hematoxylin and eosin (H&E) using routine methods. Commercially available primary antibody to human Ki-67 (ZSGB-BIO, Beijing, China) was used for Ki-67 staining. After heat-induced antigen retrieval, formalin-fixed and paraffin-embedded tumor tissue sections were stained with primary antibody overnight at 4°C. The slides were subsequently incubated with ImmPRES anti-mouse Ig (Vector Laboratories, Burlingame, CA) at room temperature for 30 min, stained with peroxidase substrate 3, 3′-diaminobenzidine chromogen (Vector Laboratories), and finally counterstained with hematoxylin. TUNEL staining was assessed using In Situ Cell Death Detection Kit (POD) (Roche, Mannheim, Germany) according to the manufacturer's instructions.

### Supplementary information

Supplementary Information include 3 Tables and 1 Figure could be found with this article online.

## Supplementary Materials


