# Supplementary material for: Discovery and characterization of a novel potent type II native and mutant BCR-ABL inhibitor (CHMFL-074) for Chronic Myeloid Leukemia (CML)
Source: Oncotarget. 2016 Jun 14;7(29):45562–74. doi: 10.18632/oncotarget.10037 (PMC5216742; doi:10.18632/oncotarget.10037)
Supplement: Supplementary file 1 [file oncotarget-07-45562-s001.pdf]

Discovery and characterization of a novel potent type II native and mutant BCR-ABL inhibitor (CHMFL-074) for Chronic Myeloid Leukemia (CML)

Supplementary Materials

```
CLUSTAL O(1.2.1) multiple sequence alignment


1T46:A|KIT      GNNYVYIDPTQLPYDHKWEFPRNRLSFGKTLGAGAFGKVVEATAYGLIKSDAAMTVAVKM
PDGFRA          ------------------------LVLGRVLGSGAFGKVVEGTAYGLSRSQPVMKVAVKM
PDGFRB          ------------------------LVLGRTLGSGAFGQVVEATAHGLSHSQATMKVAVKM
                                        * :*:.**:****:***.**:** :*: .*.*****

1T46:A|KIT      LKPSAHLTEREALMSELKVLSYLGNHMNIVNLLGACTIGGPTLVITEYCCYGDLLNFLRR
PDGFRA          LKPTARSSEKQALMSELKIMTHLGPHLNIVNLLGACTKSGPIYIITEYCFYGDLVNYLHK
PDGFRB          LKSTARSSEKQALMSELKIMSHLGPHLNVVNLLGACTKGGPIYIITEYCRYGDLVDYLHR
                ** :*: :*::*******::::** *:*:******** .**  :***** ****:::*::

1T46:A|KIT      KRDSFICSKTSPAIMEDDELALDLEDLLSFSYQVAKGMAFLASKNCIHRDLAARNILLTH
PDGFRA          NRDSFE--------------GLTLLDLLSFTYQVARGMEFLASKNCVHRDLAARNVLLAQ
PDGFRB          NKHTFP--------------VLSYMDLVGFSYQVANGMEFLASKNCVHRDLAARNVLICE
                ::.:*                *   **:.*:****.** *******:********:*: .

1T46:A|KIT      GRITKICDFGLARDIKNDSNYVVKGNARLPVKWMAPESIFNCVYTFESDVWSYGIFLWEL
PDGFRA          GKIVKICDFGLARDIMHDSNYVSKGSTFLPVKWMAPESIFDNLYTTLSDVWSYGILLWEI
PDGFRB          GKLVKICDFGLARDIMRDSNYISKGSTFLPLKWMAPESIFNSLYTTLSDVWSFGILLWEI
                *::.*********** .****: **.: **:*********: :**  *****:**:***:

1T46:A|KIT      FSLGSSPYPGMPVDSKFYKMIKEGFRMLSPEHAPAEMYDIMKTCWDADPLKRPTFKQIVQ
PDGFRA          FSLGGTPYPGMMVDSTFYNKIKSGYRMAKPDHATSEVYEIMVKCWNSEPEKRPSFYHLSE
PDGFRB          FTLGGTPYPELPMNEQFYNAIKRGYRMAQPAHASDEIYEIMQKCWEEKFEIRPPFSQLVL
                *:**.:*** : ::. **: ** *:** .* **  *:*:** .**: .   ** * ::

1T46:A|KIT      LIEKQISESTNHI
PDGFRA          IVENLL-------
PDGFRB          LLERLL-------
                ::*. :
```

Supplementary Figure S1: Sequence alignment of PDGFR α/β with cKit.

Supplementary Table S1: Kinomewide selectivity profiling of CHMFL-074 with DiscoveRx's KinomeScan™ technology. See Supplementary_Table_S1

Supplementary Table S2: Potential targets revealed by KinomeScan™ Profiling bear the % control number less than 1 at 1 µM concentration of CHMFL-074

Kinases	% Control
ABL1(E255K)-phosphorylated	0.3
ABL1(H396P)-nonphosphorylated	0.15
ABL1(H396P)-phosphorylated	0.3
ABL1(M351T)-phosphorylated	1
ABL1(Q252H)-nonphosphorylated	0.75
ABL1(Q252H)-phosphorylated	0.4
ABL1(Y253F)-phosphorylated	0.3
ABL1-nonphosphorylated	0.25
ABL1-phosphorylated	0.2
ABL2	0.8
BLK	0
CSF1R	0.2
DDR1	0.6
DDR2	0
KIT(L576P)	1
KIT(V559D)	0.45
LCK	0.2
LOK	0.1
PDGFRB	0.2
RET	0.05
RET(M918T)	0.15

Supplementary Table S3: Crystal structure data collection and refinement statistics

Structure and PDB ID	ABL1/CHMFL-074 complex 5HU9
**Data collection***	
Space group	$C222_1$
Cell dimensions	
a, b, c (Å)	37.8, 108.4, 146.3
α, β, γ (°)	90.0, 90.0, 90.0
Resolution (Å)	50–1.53 (1.56–1.53)
$R_{p.i.m.}$(%)**	2.8 (35.8)
I/σ	27.1 (2.0)
Completeness (%)	97.7 (89.6)
Redundancy	12.0 (10.1)
**Refinement**	
Resolution (Å)	43.56–1.53
No. reflections	44775
$R_{wor}k/R_{free}$	0.185/0.196
No. atoms	
Protein	2251
Ligand/ion	135
Water	365
B-factors	
Protein	28.4
Ligand/ion	31.5
Water	42.8
R.m.s. deviations	
Bond lengths (Å)	0.023
Bond angles (°)	1.689
Ramachandran Plot	
Favoured region	97.50%
Allowed region	2.50%
Outliers	0.00%
